# Experiencing and responding to chronic cancer‐related fatigue: A meta‐ethnography of qualitative research

**DOI:** 10.1002/pon.5213

**Published:** 2019-09-10

**Authors:** Tom I. Bootsma, Melanie P.J. Schellekens, Rosalie A.M. van Woezik, Marije L. van der Lee, Jenny Slatman

**Affiliations:** ^1^ Helen Dowling Institute Centre for Psycho‐Oncology Scientific Research Department Bilthoven The Netherlands; ^2^ Tilburg University School of Humanities and Digital Sciences Culture Studies Department Tilburg The Netherlands

**Keywords:** cancer, chronic cancer‐related fatigue, experience, meta‐ethnography, oncology, qualitative research

## Abstract

**Objective:**

One of the most prevalent and disrupting symptoms experienced by cancer patients is chronic cancer‐related fatigue (CCRF). A better understanding of the chronic nature of CCRF can provide valuable insights for theory and practice. The purpose of this meta‐ethnography was to derive an overarching interpretative narrative on patients' experiences and responses to CCRF.

**Methods:**

We conducted a comprehensive systematic literature search in five databases (05‐03‐2018). In addition, papers from reference lists were retrieved. Two researchers independently screened the papers for eligibility and appraised quality (CASP‐criteria). We followed the seven phases of meta‐ethnography to extract, translate, and synthesise first‐order constructs (ie, patients' views) and second‐order constructs (ie, authors' views) from the selected studies into third‐order constructs (ie, new interpretations).

**Results:**

Of the 1178 collected articles, 16 articles were included. Through synthesis, a new figure of six interrelated third‐order constructs was developed: *(1) embodied experience* entails the dominating presence of the body; *(2) (mis)recognition* includes lack of recognition of CCRF by patients, relatives, and health providers; *(3) small horizon* describes a resultant narrowed world; *(4) role change* encompasses adopting other life roles; *(5) loss of self* refers to the impact on one's identity; *and (6) regaining one's footing* describes the struggle against CCRF, adaptation to CCRF and finally acceptance of a “new normal with CCRF.”

**Conclusion:**

A new embodiment figure of CCRF with social (eg, (mis)recognition), spatial (eg, small horizon) and temporal dimensions (eg, regaining one's footing) was developed. This figure can help professionals to recognise CCRF, inform patients, and personalise treatment.

## 1 BACKGROUND

Fatigue is one of the most prevalent and disrupting symptoms experienced by cancer patients.^1^, ^2^, ^3^, ^4^, ^5^, ^6^ The National Comprehensive Cancer Network (NCCN) has defined such fatigue as “a distressing, persistent, subjective sense of physical, emotional, and/ or cognitive tiredness or exhaustion related to cancer or cancer treatment, that is not proportional to recent activity and interferes with usual functioning”.^7^ In approximately 25% of patients, this fatigue is defined as chronic cancer‐related fatigue (CCRF), because it persists for months to years after treatment completion.^6^, ^8^, ^9^ CCRF is often accompanied by distress, interferes with daily activities and social relationships, and limits patient's quality of life.^10^, ^11^, ^12^, ^13^ In addition, CCRF is an important public health issue that incurs medical and societal costs.^14^, ^15^

The patient perspective and experiential knowledge of qualitative studies provide a richness and depth that complements quantitative studies.^16^ As such, it can help to gain a better understanding of what CCRF entails and to provide insight into how to best treat these patients.^17^, ^18^ Several qualitative studies have examined patients' experiences with fatigue in different cancer populations. For example, Levkovich et al conducted interviews amongst breast cancer patients after chemotherapy and two main themes emerged: “Being imprisoned in the body of an 80‐year‐old” and “Family's bear‐hug”.^19^

Scott et al (2009) analysed patients' quotations and published a systematic review of qualitative studies focusing on fatigue across the cancer trajectory.^20^ They concluded that patients often describe how fatigue affects their lives, rather than the sensations of fatigue.^20^ Moreover, “tiredness” does not capture the multidimensional character of the patient's fatigue experience.^20^ So far, most qualitative reviews^20^, ^21^ have focused on experiences of fatigue in the acute phase of treatment, whilst the long‐term chronic nature of this condition has remained less well understood. Therefore, a more detailed and up‐to‐date description of the chronicity of post‐treatment fatigue is required.

Although previous qualitative research on CCRF provided valuable insights, the increasing number of new studies made it difficult for researchers and clinicians to appreciate which insights were most useful for further research or clinical practice. Thus far, qualitative reviews have performed comparative and aggregative analyses.^20^, ^21^ In order to personalise treatment for CCRF, a more interpretative approach to analyse individual studies is needed. Therefore, we conducted a meta‐ethnography to create an overarching interpretative narrative, whilst maintaining the integrity of the individual studies.^22^, ^23^, ^24^, ^25^ This meta‐ethnography focused on how patients experience and respond to CCRF.

## 2 METHODS

### 2.1 Design

This study was based on Noblit and Hare's interpretative and iterative approach for conducting a meta‐ethnography, modified with knowledge from practical examples in health care whilst utilising the eMERGe guidance for reporting.^24^, ^25^, ^26^, ^27^, ^28^ The seven phases used in this approach were followed: (a) formulating the research question; (b) searching and screening studies to decide what is relevant; (c) reading studies to become as familiar as possible with the conceptual content (d) determining how studies are related through coding and comparing conceptual content; (e) translating the studies into one another; (f) synthesising the translations; and (g) expressing the synthesis in a figure for use by health professionals.^23^, ^24^

### 2.2 Search method and identification of the studies

Systematic comprehensive literature searches in Pubmed/ Medline, EMBASE, CINAHL, PsycINFO, and Web of Science were conducted by TB (05‐03‐2018) (Table S1 for STARLITE criteria). Studies were included when they met the following eligibility criteria: (a) explored the experiences of adult patients with CCRF post‐treatment; (b) used qualitative methodology to analyse data; and (c) were published in English or Dutch. Table S1 shows exclusion criteria. Studies were first screened on title and abstract (T.B. and R.W. individually), followed by full‐text screening (T.B. and M.S. individually). Discrepancies between selections were discussed until consensus was reached.

### 2.3 Quality appraisal

The selected papers were reviewed individually by T.B. and M.S. using the 10‐item Critical Appraisal Skills Programme (CASP) checklist to assess whether the results were valid and reliable.^29^ Whilst the use of quality criteria is widely debated, we chose this commonly used appraisal tool to gain insight into the content and quality of the current evidence. The intention was not to reach consensus in scores on the CASP questions. The answer to each CASP question was counted by each researcher as fully addressed (three points), partially addressed (two points), and not addressed (one point), leading to a score ranging from 10 to 30.

### 2.4 Data‐analysis and synthesis

After reading and re‐reading the included papers, first‐order (ie, patients' views on experiences) and second‐order constructs (ie, authors' interpretations of patients' views on experiences) of the selected studies were identified and listed with the help of the software programme, MaxQDA 2018 (release 18.1.1). First‐order constructs involve patients' understandings (quotes) of CCRF and were extracted from the results section of the selected paper.^23^, ^24^ Second‐order constructs involve the authors' interpretations (themes and concepts) of patients' understanding of CCRF and were usually extracted from the discussion and conclusion sections of the selected paper.^23^, ^24^ We created third‐order constructs (ie, new interpretations) by translating and synthesising these first‐order and second‐order constructs.

Different constructs between and within studies were compared. As the content showed primarily similarities, a reciprocal translation was applied. Inconsistencies were handled by refutational translation. For example, the third‐order construct (mis)recognition encompassed both misrecognition and recognition. Thematic analysis started by listing constructs in conceptual categories of the most recently published and conceptually rich paper.^19^ T.B. and R.W. independently listed the first‐order and second‐order constructs of four different papers.^19^, ^30^, ^31^, ^32^ Subsequently, the construct list of these papers was compare to reach consensus, creating a final list with which the other papers were inductively analysed. M.S. supervised this listing process.

Group discussions were organised twice with a multidisciplinary team of T.B. (PhD student, MSc Medicine), M.S. (Postdoc researcher Psycho‐Oncology), R.W. (Research assistant, Master student Behavioural Science), M.L. (Senior Researcher Psycho‐Oncology and Psychologist treating patients with CCRF), and J.S. (Professor in Medical Humanities). T.B., M.S., M.L., and J.S. have extensive experience in conducting qualitative research in psycho‐oncology. During our team discussions, we reflected on our own position and perspective to diminish bias.

Group discussions were based on merging conceptual categories (subthemes). Patients' experiences and responses to CCRF were approached as a whole, and descriptions of temporal changes in these experiences and responses were taken into account. During the last group discussion, we adopted a line of argument synthesis and visualised the relations between third‐order constructs (meta‐themes) to complete the overarching narrative.^23^, ^24^ We cross‐checked to determine whether the individual studies fitted these meta‐themes.

## 3 RESULTS

### 3.1 Searches and study characteristics

The searches resulted in a total of 1178 articles (Figure S1). After deduplication, title/abstract, and full‐text screening, 1159 articles were excluded. Concurrence was reached to exclude four extra articles, based on article type (brief report)^33^ or deductive qualitative methodology (secondary data‐analysis or testing a model).^34^, ^35^, ^36^ Finally, we included 16 studies in the meta‐ethnography.^19^, ^30^, ^31^, ^32^, ^37^, ^38^, ^39^, ^40^, ^41^, ^42^, ^43^, ^44^, ^45^, ^46^, ^47^, ^48^ Study characteristics are summarised in Table S2. In total, 705 patients, particularly women (29%‐100% in reported study samples), were included in these studies. Patients of different ages, cancer types and stages (mainly breast cancer), and treatment phases participated. Four studies compared cancer patients to healthy subjects^39^, ^40^ or other patient groups.^38^, ^41^ Data of healthy subjects and other patient groups were excluded from analysis. Some studies were part of larger projects^31^, ^44^, ^45^ or did not have qualitative data collection methods (eg, blogs).^31^, ^44^, ^47^, ^48^ Quality appraisal by CASP criteria resulted in varying quality (scores 16‐28 points) (Table S3). All studies clearly described the aims of the research and rationale for the qualitative methodology.^19^, ^30^, ^31^, ^32^, ^37^, ^38^, ^39^, ^40^, ^41^, ^42^, ^43^, ^44^, ^45^, ^46^, ^47^, ^48^ The majority, however, did not adequately consider the relationship between authors and participants.^31^, ^32^, ^37^, ^39^, ^40^, ^41^, ^42^, ^43^, ^44^, ^45^, ^46^, ^47^ None of the studies were excluded based on quality appraisal.

### 3.2 Meta‐themes

The first‐order (quotes in *italics*) and second‐order (themes/concepts in normal font) constructs were translated in six new meta‐themes: embodied experience, (mis)recognition, small horizon, role change, loss of self, and regaining one's footing (Table S4). Individuals differed in their experiences and responses. That is, not all individuals had all experiences/ responses described in the meta‐themes.

1. Embodied experience

Patients' descriptions of CCRF were embodied, meaning that their experiences were predominated by bodily sensations or bodily symptoms,^38^, ^39^, ^40^, ^44^, ^45^ such as *“heavy limbs”*,^39^ *“legs like jelly or wobbly legs”*,^39^ *“feeling weak”*,^19^, ^31^, ^45^, ^46^ or *“paralysis”*.^42^ CCRF was often described as a whole body experience: *“the body is worn‐out”*,^39^, ^40^, ^46^ *“physically exhausted”*,^19^, ^39^, ^45^ *“body doesn't want to go on”*,^30^ or *“body cannot heal, nor can it function well”*.^46^ One patient commented that her body could not carry her:

“It's like the body can't … carry you. It's a real weakness … a bad feeling. And it's simply … like you don't have any energy, no energy for anything. Nothing, like you're … you're like a zombie; you just sit there … like a couch potato” F (Female), 67 (age), breast cancer (cancer type)^19^ (p6).

Whilst several studies reported cognitive and affective symptoms of CCRF,^19^, ^30^, ^31^, ^32^, ^38^, ^39^, ^40^, ^43^, ^45^, ^46^, ^47^ patients often described these symptoms as part of the body. For example, the metaphor *“the brain is out of function”* was commonly used to describe a cognitive sensation of tiredness in the head.^30^, ^40^, ^41^

Consequently, patients reported they became more aware of their bodies in various ways: *“the fatigue takes over every aspect of the body”* 19; *“the feeling that the body dominated the mind”* 39; or *“the whole body is shattered”*.^40^ Other patients described CCRF as *“the betrayal of the body”* and *“trapped in an old, sick body”*.^19^, ^39^, ^41^

Patients' experiences revealed that physical and mental symptoms of CCRF are strongly interrelated.^37^, ^39^, ^40^, ^45^, ^46^, ^47^ One patient described this relationship:

“Fatigue is a physical experience but there is also something psychological; if one thinks of being a death candidate, it's not cheering you up” N/ A (Identification Not Available)^40^ (p87).

- (Mis)Recognition

As CCRF is invisible, and healthy individuals also experience tiredness, patients felt it was difficult to explain to others how intense CCRF is.^30^, ^32^, ^37^, ^38^, ^39^, ^40^, ^43^, ^44^, ^46^, ^47^ Consequently, others did not recognise patients' experiences^30^, ^42^, ^43^, ^44^, ^46^, ^47^:

“Because I look so much better than when I was having treatment, people think that I am back to prior fitness” F, N/ A^44^ (p153).

Patients also experienced being misunderstood by their friends and family.^30^, ^43^, ^44^, ^46^, ^47^ One patient described how the family failed to remember the fatigue*:*

“My family is pretty good but they can easily forget how tired I can get as this is not obvious” F, N/ A^44^ (p153).

Patients often experienced a conflict between their own needs and the expectations around them.^19^, ^47^ Patients felt pressured to cease being sick and move on^44^ and, as a result, did not feel supported by their family and friends.^19^, ^42^, ^43^, ^47^ One patient reported about her unsupportive mother:

“My mum basically accused me of not trying hard enough to get over it” F, N/ A^44^ (p156).

Moreover, in some papers, patients indicated that health care professionals did not recognise CCRF.^42^, ^44^ Because of a lack of objective medical parameters, health care professionals approached CCRF as a problem with *“no symptoms, no cause and no cure”*.^44^ Therefore patients had negative experiences in communicating about CCRF with health care professionals and sometimes had the feeling they were not being heard or taken seriously.^44^, ^45^ This was primarily caused by a lack of awareness amongst health care professionals.^32^, ^44^ Patients voiced that for health care professionals CCRF is *“the least worrying side effect”* of cancer treatment, and as a result, *“comes at the bottom of the list”*.^32^ This resulted in the management of CCRF being a low priority.^32^

Patients often did not get the right information about what they could expect in terms of CCRF after treatment.^32^, ^44^, ^46^ Patients who were informed ahead of time appreciated the information that fatigue was a common experience and were then able to legitimise their fatigue.^32^, ^43^ One patient explained how better information improved recognition of CCRF:

“This material has helped a lot, it has given me good advice and especially I felt reassured that fatigue was not absurd or unusual but that others suffer from it as well” N/ A^32^ (p105).

Patients were reluctant to talk about their fatigue in general which contributed to the “misrecognition” of fatigue.^43^, ^46^, ^48^ As patients were often not informed about CCRF by health care professionals, they did not recognise their symptoms of CCRF.^19^, ^30^, ^44^, ^46^ One patient described the misrecognition:

“I have not been told that I have fatigue but I seem to be living with it‐ it varies a lot but I do believe it interferes with my daily life and the sense of needing to ‘keep going' because ‘it must be all in my head' because I've not really had any diagnosis, and this makes me feel worse when I am tired, because I feel silly” N/ A^44^ (p154).

As a result of not being informed and prepared by health care professionals for experiencing CCRF after cancer treatment, patients had pessimistic beliefs, for example CCRF is: *“untreatable”*,^45^ *“inevitable”*,^43^, ^45^, ^47^ *“sign of decline in health”*,^30^ *“it is a sign of not being successfully recovered from cancer or cancer recurrence”*,^47^ *“it indicates the body cannot heal nor function well”*,^46^ *“it elicits concerns about survival”*,^31^ or *“caused by the unknown”*.^31^ Patients felt embarrassed, which further limited their willingness to discuss CCRF with others.^19^, ^39^, ^43^ Patients experienced a gap between expected “life after illness” and reality.^19^ Furthermore, patients could lose control of their own story, due to the lack of conceptualisation of the CCRF they felt in a “limbo between sickness and survivorship”.^44^ One patient expected the future to be limited by their fatigue:

“I've settled in my mind that I'm going to get worse; I expect it to happen gradually, and I expect one of the ways it will show is that I will be more tired than I am now” N/ A^37^ (p114).

On the other hand, in some studies, patients recognised fatigue as *“abnormal”* and *“pathological”* when it persisted after treatment^39^, ^41^, ^42^ and sought medical help.^19^, ^30^, ^31^, ^32^, ^43^, ^44^, ^45^, ^46^, ^48^ For some patients, CCRF had become “an illness with its own rights”.^39^, ^42^

Patients with optimistic beliefs expected a future of normal pre‐diagnosis levels of fatigue^37^ or convinced themselves to be okay.^32^ One patient reported:

“I don't think there's going to be any problem in the future” N/ A^37^ (p114).

- Small horizon

The physical, emotional, cognitive, and social consequences and limitations of CCRF resulted in a “small horizon” for some patients. It reduced their freedom and narrowed their scope of abilities. Patients were unable to regain their precancer activity level.^31^, ^37^, ^38^, ^41^, ^43^ The various dysfunctions and inabilities included: reading,^39^, ^41^ getting out of bed,^19^, ^31^ moving,^42^, ^45^ walking,^39^, ^40^, ^45^ climbing stairs,^39^ carrying out household tasks,^38^, ^39^ praying,^30^ working,^30^, ^32^, ^42^ and socialising.^19^, ^30^, ^31^, ^32^, ^47^ Thus, CCRF influences all kind of aspects of daily life and is seen as an “obstacle in daily life”.^19^, ^30^, ^37^, ^38^, ^42^, ^44^

These dysfunctions and inabilities were often accompanied by feelings of isolation or nonbelonging.^31^, ^39^, ^44^ Patients used the following metaphors to describe their nonbelonging: *“gradual submersion”*, *“drowning”*, and *“rising water”.* 39 Some patients wanted to be left alone and face their fatigue by themselves.^19^, ^43^, ^44^

Because of the struggle with dysfunctions and inabilities, the experience of CCRF had a major impact on patients' motivation to do things.^30^, ^38^, ^39^, ^41^ This loss of interest in enjoyable things and in life in general^30^, ^38^, ^39^, ^46^ contributed to the perception of “small horizon.” One woman described this as a state of lethargy:

“I don't sleep well. I lie awake for hours at night, and in the morning, I drag myself out of bed, straggle around taking endless catnaps, living in a state of lethargy, where just watching the clouds go by is my only interest. No reading, no pleasure in doing anything whatsoever, especially all those activities I so loved before I became ill and tired … and in the evening I never feel like going to bed. On the other hand I just fall asleep when and where I shouldn't in the day” F, 62, leiomyosarcoma uterus^39^ (p299).

- Role change

The patients' suffering caused by CCRF interfered with responsibilities in daily life and performing life roles,^19^, ^37^, ^45^, ^46^ because of their inability to function normally at home and work.^38^, ^41^, ^42^, ^43^, ^47^ Most patients became dependent upon others, who took over activities.^19^, ^31^, ^39^, ^43^ One problem related to suffering from CCRF and becoming dependent was the impact on the family role and family system,^19^, ^30^, ^31^, ^37^, ^41^, ^43^, ^46^ which could result in stress and conflicts in families.^19^, ^43^ One woman described her role change in becoming a “limited mother”:

“I let go, you know … it's okay. It doesn't mean anything. True, you were used to running things … at first, it's some kind of contradiction, it's … it's a shock. You manage everything, then suddenly you feel … limited. Even your young child is bringing you a glass of water” F, 43, breast cancer^19^ (p6).

Suffering from CCRF and becoming dependent also affected the partner role.^19^, ^31^ In this example, the partner was supportive, and the patient was accepting help:

“I actually felt like I was being led like a little girl … whose father is leading her [by the hand]” F, 43, breast cancer^19^ (p7).

Partners could also be less supportive or even unsupportive. Patients indicated they experienced no closeness in their relationships, or even failure of marriage.^19^, ^31^ One patient described how her partner did not support her:

“He asked me if I could help him with something … I said—Me help you?! You need to help me! How can I help you? I can't … it was really difficult” F, 65, breast cancer^19^ (p7).

Patients' suffering from CCRF and losing their independency had a major impact and caused distress in their social support network.^19^, ^30^, ^31^, ^32^, ^38^, ^40^, ^41^, ^42^, ^43^, ^44^, ^46^ Roles changed from helper to being helped:

“I'm the type of person, when someone's ill or needy, or a friend needs something … I'm there right away … reporting for duty. Immediately. There's no way that someone needs something and I won't turn the world upside down to help them and … what happened actually, with my illness, is that … it's as if everyone I've ever helped, everyone I've spent time with … came to repay me” F, 41, breast cancer^19^ (p7).

Furthermore, person's social well‐being was disrupted,^31^ because of their inability to attend activities in community life.^46^

People with CCRF chose to cut back on their workload, to quit work or to retire from their work to have more time for themselves.^30^, ^31^, ^37^, ^42^, ^47^ One patient described that suffering from CCRF prevented him from returning to work:

“I was determined to go back to work as soon as I possibly could. I mean I haven't a particularly hard job, but then I did it for a week and just had to, just couldn't do it anymore, and it really shocked me, that I felt so tired, so fatigued…so I think it would probably be quite handy to just make people aware of the fact that even though you are feeling quite well that the fatigue is going to get you, possibly will get you, and may well mean that you can't work” F, N/ A^32^ (p106).

- Loss of self

People described their change as that from being a healthy person to being a patient with cancer, suffering a different and persistent fatigue compared with the fatigue they experienced precancer.^30^ Patients described different feelings related to *“loss of self”*, for example a lack of control,^19^, ^30^, ^31^, ^32^, ^38^, ^39^, ^44^ loss of confidence,^39^, ^41^, ^44^ worthlessness,^31^, ^41^ indecisiveness,^30^, ^40^ and uncertainty about the ability to cope with fatigue.^19^, ^30^, ^31^, ^38^, ^39^, ^40^ Patients also experienced a loss of purpose in life, for example, by losing their career plans.^31^, ^44^ In some cases, patients expressed an awareness of the finite nature of life:

“Hanging on to life by a thread of cotton”, “walking along the razor's edge” or “along a tight rope” N/ A^39^ (p304).

As a result of these feelings, including their small horizon and dependency, patients described a loss of self.^39^, ^44^

“I was completely exhausted and in despair, living in a state of “absence”, with regard to myself. My fatigue no longer served as an alert to the gravity of my condition. Rather, I came to tolerate it, unaware of the dangers due to my loss of lucidity. I felt ashamed of my powerlessness to face up to my fatigue, totally lost like a compass unable to find the North” F, 62, leiomyosarcoma uterus^39^ (p310).

Patients grieved for the person they had been and realised they could not go back to their “old self” before the cancer.^37^, ^38^, ^39^, ^44^ One patient quoted:

“I'm obviously not the man I was” N/ A^37^ (p114).

Other patients commented on their changes in identity, for example from an active to an inactive person.^30^, ^39^, ^45^ A frequently used metaphor for the change in identity, emphasising the embodied experience of CCRF, was feeling an old sick person inside of them^19^, ^44^, ^45^:

“I feel like I'm in the body of an 80‐year‐old. That theoretically I know what my capabilities are, but … in reality … it's different. It's two different things. You know what you can do. But on the … on the other hand, you don't. Now, it's … exactly like an 80‐year‐old. Every single thing is ‘a project'. I cook, I need to sit down and rest. Ten minutes, yes? Prepare something, need to sit down and rest. These are things that … it's like being trapped in the body of an 80‐year‐old” F, 34, breast cancer^19^ (p5).

- Regaining one's footing

Regaining one's footing involves different successive approaches (struggling, adaptation, and acceptance) in the process of finding a “new normal” with CCRF. The individual's perception of fatigue varied according to their personality, vulnerability, ways of coping with stressors, general wellbeing, and social support.^31^, ^38^, ^40^

First, several patients described how they struggled against CCRF. They tried to fight it, continued their life roles, tried to fix it, distract themselves, and even concealed their CCRF.^19^, ^31^, ^32^, ^40^, ^43^, ^44^, ^45^, ^47^, ^48^ One patient told about her fight against CCRF:

“I was very tired … I was tired, but my goal—my goal was to get through it as fast as I could. I pushed myself, I pushed myself, I pushed myself. Yeah, I was tired. I mean, I would be at work, I would have to put my head down or my sister used to work with me and I would tell her, ‘You know what? I'm just going to go take a nap.' But I pushed myself, I pushed myself and I, I never gave up my heels” F, N/ A^45^ (p11).

After a phase of struggling, several patients used more adaptive responses to monitor and pace their activities. Monitoring of activities was used by patients to regain control of CCRF by exercising,^31^, ^39^, ^43^, ^47^, ^48^ lifestyle changes,^30^, ^31^, ^43^, ^47^, ^48^ keeping a diary,^32^, ^48^ or building their lives entirely on their fatigue experience.^42^ One patient mentioned changing to a more regular lifestyle:

“I have a very orderly and structured lifestyle. I don't do anything which has not been planned. Every day I get up at 9 o'clock, I read, I do my shopping; I always eat at 1 p.m. before having a nap until 4, then I watch a bit of TV, I eat at 6, lie down until 8, watch TV with my husband, then go back to bed at 10. It's a very regular life, but it suits me” F, 62, Non‐Hodgkin lymphoma^42^ (p32).

Several patients monitored their CCRF by being vigilant, recognising and setting boundaries, listening to personal needs, and listening to their bodies.^19^, ^39^, ^47^, ^48^ One patient explained:

“Fatigue taught me to listen to my body which is now so fragile” F, 48, Acute Lymphoblastic Leukaemia^39^ (p310).

CCRF patients could pace themselves in different ways. Some patients focused on stopping doing an activity: having a break,^40^ sitting or lying down to rest or sleep,^31^, ^39^, ^40^, ^42^, ^45^, ^47^, ^48^ resting and waiting for it to pass,^19^, ^31^, ^37^, ^39^, ^43^ and avoiding physically straining activities.^30^, ^42^ Other patients re‐shaped their daily lives: living their life day by day,^38^, ^43^ planning,^19^, ^30^, ^42^, ^43^, ^48^ prioritising,^19^, ^30^, ^43^, ^48^ reorganising life and work,^19^, ^30^, ^42^, ^43^, ^47^ and balancing activities.^19^, ^46^ One patient commented on how she lived at a slower pace:

“If I'm more tired, it's not the end of the world, so I'll rest and do less. It's not the end of the world and it's possible to live at a slower pace than I was used to doing” F, 60, breast cancer^19^ (p6).

In this quote, a patient reorganised life by quitting work because of CCRF:

“I was a very active person, and I've gone from a very active life to … if I overexert, I'll tire myself out. I can't work for a couple reasons. First, the bank won't let me come back without a full medical release. Second, I wouldn't be able to do the job. You just have to adjust your lifestyle, and try to get the control back” M (Male), 51, gastro‐esophageal cancer^30^ (p92).

Finally, some patients accepted CCRF as a “new normal,” by internalising the current changes, trying to see positive aspects and finding balance.^19^, ^31^, ^37^, ^43^, ^47^, ^48^ One patient shared how she changed her attitude to accept her situation:

“It's all about attitude really … in altering what you can, accepting what you can't” N/ A^37^ (p114).

As part of finding a “new normal” patients explored and reframed meaning by naming or assigning a cause to CCRF to give it a place in their life.^30^, ^31^, ^39^, ^44^ Some patients were informed by their health care professionals to set goals and visualise strategies to overcome CCRF, to legitimise fatigue and get support from their family members.^32^, ^42^, ^43^, ^44^, ^46^, ^48^ One patient described the advantage of legitimisation of fatigue:

“It is a matter of legitimisation to experience fatigue as a usual experience” N/ A^32^ (p105).

#### 3.2.1 Relations of meta‐themes

**Figure 1:**
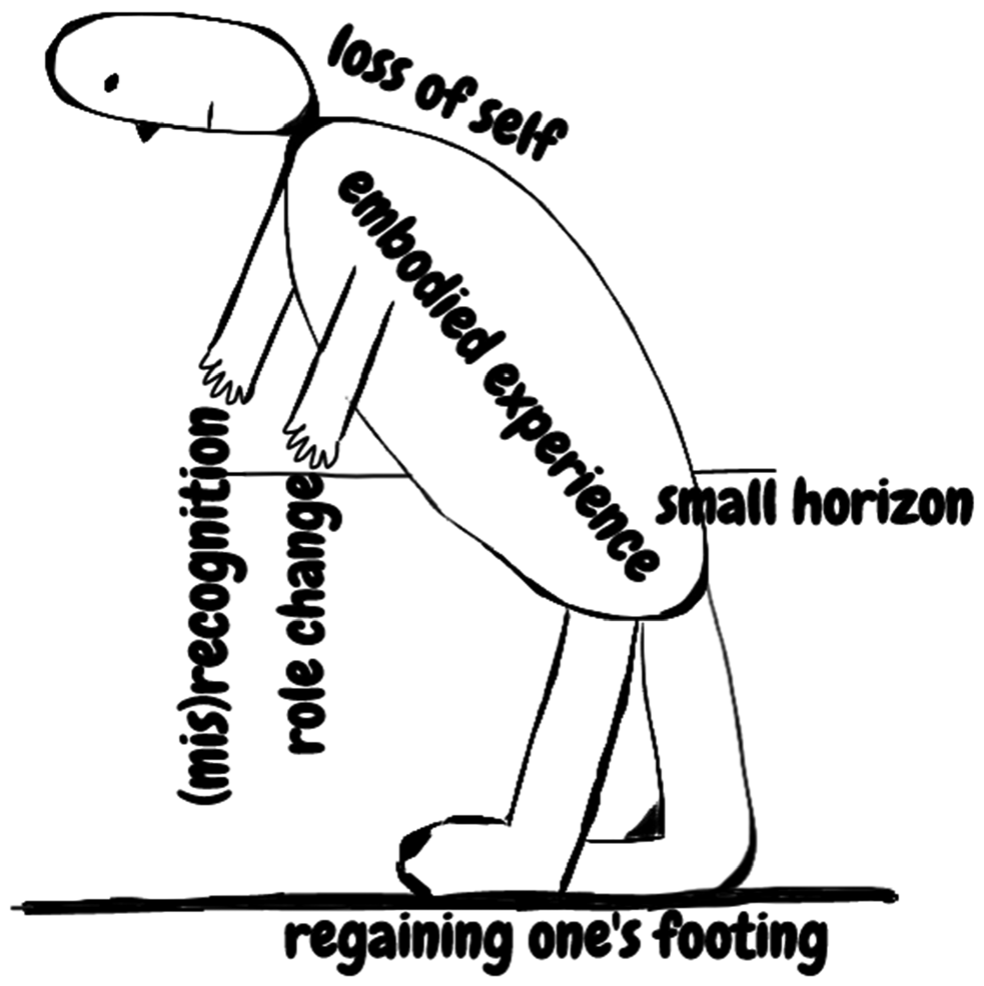
Embodiment of CCRF

The six meta‐themes we identified are interrelated. Central to all themes is the phenomenon of embodiment. Acknowledging that humans are primarily embodied beings—instead of composites of (disembodied) mind and body—embodiment refers to the fact that action, perception, orientation, and cognition are based on our being embedded in a certain time and place, and social situation through our bodies. Figure 1 captures the embodied dimension of CCRF. Fatigue is experienced in one's (entire) body, going together with loss of self. Role change and (mis)recognition are symbolised by the hands, “embodied tools” for intentional actions within one's social context. The small horizon behind the body represents the narrowed world. Regaining one's footing is symbolised by the feet who seek to regain a new “optimum equilibrium” in one's world (49 p.109). How the various aspects of embodiment relate to one another depends on the individual patient. The following quote, for example, shows how role change, small horizon and (mis)recognition are interrelated in this respondent:

“I am at the age where people around me go out, party and have fun all the time. That's what college students do! … .. but I can't. Nobody understands that” N/ A^47^ (p4).

The embodied structure of CCRF involves social (eg, (mis)recognition and role change), spatial (eg, small horizon), and temporal (eg, loss of self and regaining one's footing) dimensions.

## 4 DISCUSSION

### 4.1 Findings of synthesis

To our knowledge, this interpretative review is the first meta‐ethnography based on 16 qualitative studies that unravelled patients' perspective on the chronic nature of CCRF. The main finding of this synthesis is the embodied structure of CCRF that can be used in clinical practice for treating patients and in research for future development of measurement. Subsequently, a new figure was developed to visualise the valuable insights into how patients experience and respond to CCRF.

Previously, the onset of fatigue by patients undergoing treatment has been linked to paying explicit attention to the body as problematic and as an object.^50^, ^51^ In accordance with these studies, our results show that the awareness of the body in a negative way dominates patients' experience and is described as “old” and “sick.” Although the human experience is always embodied, in everyday life the body is seldom a thematic object of experience, as there are less thoughts about the body and it dis‐appears from attention.^52^, ^53^ Drawing on Leder's analysis of how one's own body can be either present or absent to oneself, the term dys‐appearance (dys is a Greek prefix) is used to describe the situation when the body appears to the patient as “ill” or “bad”.^52^, ^53^

Because the body dys‐appears whilst being chronically fatigued, it becomes the focal point of attention. The body loses its taken‐for‐granted dimension.^52^ Consequently, the agency over one's body declines.^52^ From a phenomenological point of view, it is claimed that whenever one's body appears obstinately in the foreground, one's possibilities to act and to endow meaning to one's situation, also defined as “*I can,*” shrink.^54^ As a consequence of these reduced possibilities, the ability to adequately respond to the situation lessens.^54^ Regaining one's footing, by contrast, implies that one is able to resume a certain renewed taken‐for‐granted view of one's body. Patients reported that pacing and monitoring activities helped to regain such a new equilibrium. In line with previous qualitative research^50^ and meta‐ethnographies in patients with cancer,^55^ and chronic pain,^26^ these strategies help patients to improve a sense of control and harmony with their altered bodies.

The individual experience and way of responding to CCRF is not static. The embodied experience of CCRF accompanied with loss of self could dominate and represent the illness state. Regaining one's footing entails learning to relate to CCRF in a different way and can help one to return to a healthy state. Paterson's shifting perspectives model of chronic illness demonstrated that this is a dialectical, constantly shifting process, in which either the illness (eg, embodied experience) or wellness (eg, regaining one's footing) perspective is in the foreground.^56^

In comparison to the NCCN definition of CCRF (Introduction),^7^ our findings based on patients' individual experiences and responses reflect a broadened context with embodiment and the related social, temporal, and spatial dimensions. Further research should focus more on how to measure embodied experiences and responses to CCRF. Currently, the EORTC QLQ‐C30 is a questionnaire that captures some of these meta‐themes (role change, small horizon, and regaining one's footing) in the subscales on functioning (eg, Role Functioning and Emotional Functioning).^57^

### 4.2 Study limitations

A potential limitation of this meta‐ethnography is the inclusion of mixed cancer patients' samples: on‐/post‐treatment,^30^, ^31^, ^32^, ^37^, ^38^, ^39^, ^40^, ^43^, ^45^, ^48^ and curative/ advanced stages of disease.^30^, ^31^, ^38^, ^39^, ^40^, ^42^, ^45^, ^48^ Only in the case where the authors reported patients' experiences during treatment, did we exclude these quotes from analysis. For disease stages, no distinction was made during analysis. We included studies with diverse demographics, although most studies focused on white, middle‐aged, female breast cancer patients, shortly after treatment was finished.

The systematic search strategy we conducted cannot exclude the possibility that some articles were missed. We tended to include a purposive and comprehensive sample of studies that allowed conceptual saturation to connect with the aim: develop an interpretative explanation of responses to CCRF. We included a broad range of qualitative data (eg, interviews, blogs, and open‐ended questionnaires). Although some studies may have been of a bit lower quality, the content of all studies was valuable and informative to broaden the perspective on CCRF.

The starting point of the analysis was the newest and conceptually richest paper of Levkovich et al.^19^ We could not initially discern whether or not this starting point affected the interpretation of concepts of other studies. However, as we conducted inductive analysis and cross‐checked all studies afterwards, we expect the impact to be minimal.

Quality assessment revealed that one of the most frequent shortcomings was the lack of description of the relationship between researchers and participants.^31^, ^32^, ^37^, ^39^, ^40^, ^41^, ^42^, ^43^, ^44^, ^45^, ^46^, ^47^ Transparency in terms of the role of researchers and the ongoing critical reflection of their own biases is important in qualitative research, because of the level of subjectivity in this methodology. Future qualitative research should be attentive to the description of their methods section and should use, for example, the COREQ checklist.^58^ The quality of the included studies impacts on the quality of the present results. However, by having carefully applied the meta‐ethnography method, our results are considered to be reliable.

### 4.3 Clinical implications

The social, spatial, and temporal dimensions underline CCRF's complex manifestation, which, ideally, requires a person‐centred approach by health care professionals.^59^ During, as well as after, treatment, patients need information on CCRF and other side effects.^44^, ^60^, ^61^ Whilst not all educational interventions have the benefit of positively impacting the problem of fatigue, they do appear to have a moderating effect on reducing the related distress.^62^ To prevent the false understanding of CCRF amongst patients, partners, family members, and health care professionals, several improvements are needed in providing: information, knowledge base, and education. Our first impression from clinical practice is that the figure can facilitate the communication between patients and health care professionals. It can stimulate patients to discuss their problems, because they recognise their own experiences in the figure. By normalising patients' experiences, they can regain a sense of control. The figure could also help advise patients on helpful strategies to manage CCRF. For example, by first explaining embodiment to the patient, therapy could focus on softening bodily dys‐appearance and, as such, enable bodily dis‐appearance.^63^

## 5 CONCLUSIONS

In conclusion, the main finding of this meta‐ethnography is that patients' experiences and responses to CCRF are based on being embodied. Our findings emphasise the importance of informing patients to improve recognition and healthy responding to CCRF. In future qualitative studies, lived embodied experiences and helpful patterns of responding to CCRF could be further investigated in more heterogeneous samples, including more males and patients with different cancer types. To tailor treatment to the individual, insight into calculating the interrelationship between symptoms and ways of responding to CCRF is needed.^64^

## STATEMENT CONFLICT OF INTEREST

The authors have no conflict of interest to declare.

## STATEMENT REGARDING ETHICS

Ethical approval was not necessary for this study because it was a review article.

## Supporting information

Table S1: STARLITE principles applied to literature search
Table S2: Summary of study characteristics
Table S3 CASP appraisal results
Table S4 Overview of constructs (first order, second order, and third order) and theme (sub‐ and meta‐)
Figure S1: Flow diagram study selection

## Data Availability

Data sharing is not applicable to this article as no new data were created or analysed in this study.
