# Supplementary material for: Experiencing and responding to chronic cancer‐related fatigue: A meta‐ethnography of qualitative research
Source: Psychooncology. 2019 Sep 10;29(2):241–50. doi: 10.1002/pon.5213 (PMC7027742; doi:10.1002/pon.5213)
Supplement: Supplementary file 1 — Table S1: STARLITE principles applied to literature search Table S2: Summary of study characteristics Table S3 CASP appraisal results Table S4 Overview of constructs (first order, second order, and third order) and theme (sub‐ and meta‐) Figure S1: Flow diagram study selection [file PON-29-241-s001.docx]

**Table S1: STARLITE principles applied to literature search**

|  | **Criteria** | **Results** |
| --- | --- | --- |
| **S** | **Sampling Strategy** | Comprehensive, Boolean search strategy |
| **T** | **Type of studies** | Qualitative study or qualitative research or nursing methodology research |
| **A** | **Approaches** | Electronic and reference lists |
| **R** | **Range of years** | No date limits (search 05-03-2018) |
| **L** | **Limits** | Language (English/Dutch), publication type (peer reviewed journal) |
| **I** | **Inclusions and exclusions** | Inclusion: 1) explored the experiences of adult patients with CCRF post-treatment; 2) used qualitative methodology to analyse data (i.e. oral and written descriptions of experiences and/or responses to CCRF); 3) were published in English or Dutch;  Exclusion: 1) patient sample (e.g. exclusively during treatment, exclusively advanced cancer, exclusively other condition (no cancer), children and adolescents, healthcare professionals); 2) other topic (no experiences); 3) methodology (e.g. quantitative study, meta-study, test of model) or 4) article type (e.g. abstract congress, thesis, commentary, brief report). |
| **T** | **Terms used** | Boolean search strategy (and/or) with keywords and synonyms: neoplasm or cancer or tumour, fatigue or tiredness and qualitative research for heading, topic, title and abstract |
| **E** | **Electronic sources** | Pubmed/Medline, EMBASE, CINAHL, PsycINFO, Web of Science |

**Figure S1: Flow diagram study selection**

Additional records identified through other sources(reference lists)

n=44

Records identified through database searching for abstract screening

n=1,134

identification

All identified records

n=1,178

screening

Duplicates removed:

n=567

Title and abstract screened

n=611

Reason(s) exclusion: n=541

-Not topic CRF: n=507

-Not qualitative: n=199

-Article type: n=7

n=

Full-text articles assessed for eligibility

n=70

eligible

Reason exclusion: n=54

-During treatment: n=18

-Age < 18 years: n=11

-Article type: n=8

-Advanced cancer: n=6

-Other topic: n=5

-Methodology: n=4

-Other condition: n=1

-Healthcare professionals: n=1

Articles assessed for quality

n=16

included

Studies included in qualitative synthesis

n=16

**Table S2: Summary of study characteristics**

| **Study** | **N (% females)** | **Age (range/SD)** | **Cancer type (stage)** | **Treatment** | **Treatment phase (time since diagnosis/**  **treatment)** | **Methods** | **Theoretical framework** | **Analysis** | **Aim** |
| --- | --- | --- | --- | --- | --- | --- | --- | --- | --- |
| Glaus et al. (1996)  Switzerland | 20 (45%) | 66 (42-81) | Mixed (14 different cancer types) (progressive/  stable) | Chemo- and other therapy | On/post(†) | Interviews | Grounded theory | Content analysis & constant comparison | Explore fatigue (cancer/healthy subjects) and generate theory. |
| Pearce & Richardson (1996)  UK | 6 (†) | † (†) | † (†) | Chemo-therapy | On/post(†) | Interviews | Phenomenology | Method of Giorgi (1975) | Understand and describe the meaning of fatigue. |
| Ream & Richardson  (1997)  UK | 9 (88%) | 50 (29-59) | Breast, colon, non-Hodgkin’s lymphoma, breast, rectal (metastasis yes/no) | Chemo-, radio-,  therapy | On/post (>3 months since diagnosis) | Interviews | Phenomenology | Method of Moustakas (1994) | Capture a detailed description of fatigue (cancer/chronic obstructive airways disease). |
| Holley  (2000)  USA | 17 (29%) | 59 (36-75) | Breast, head & neck,  Non-Hodgkin’s lymphoma, colon, non-small cell lung cancer, melanoma, gastro-eso-  phageal, acute lymphocytic leukaemia, squamous cell perineum, squamous cell tonsil  (stage I-IV) | Chemo-, (high dose) radio-, bio-,  therapy and peripheral stem cell transplant | On/post(†) | Interviews | Symbolic interactionism | Content analysis | Describe the experiences of fatigue, the common meanings, and the impact on patients' lives. |
| Ream et al.  (2003)  UK/  Switzerland | 15 (60%) | 59 (46-73) | Breast, bowel, ovarian, lung, stomach, multiple myeloma, prostate (†) | Chemo- and other therapy | On/post(>1 year since diagnosis) | Focus groups | Thematic framework | Framework analysis | Explore patients’ views on the current resources on the management of fatigue and providing recommenda-  tions for future development. |
| Gledhill  (2005)  France | 24 (42%) | 56 (38-78) | Mixed  (local, regional and metastasis) | † | On/post (>3 months since diagnosis) | Interviews | Grounded theory | Thematic analysis | Explore differences in the representation of fatigue (cancer/ healthy subjects); identify the concepts, dimensions and terminology of (C)CRF and fatigue in healthy persons; examine strategies used in illness and health to overcome fatigue. |
| Bennet et al.  (2007)  Australia | 16 (100%) | 56 (43-71) | Breast (stage I-II) | † | Post (>6 months since treatment) | Focus groups | Grounded theory | Multistage process: coding of symptom concepts; interpreta-tion of data and axial coding | Describe characteristics of fatigue (cancer/CFS). |
| Rosman  (2009)  The Netherlands | 12 (100%) | 46 (25-62) | Breast, Hodgkin’s lymphoma,  colon, non-Hodgkin’s lymphoma, multiple myeloma, brain tumour (metastasis yes/no) | Surgery, chemo-, radio-,  therapy, marrow bone trans-  plantation | Post (>7 years since diagnosis) | Interviews | † | Thematic analysis | Describe how the symptom can be experienced as problematic and detail coping strategies that individuals use to live with it. |
| Tsai et al.  (2010)  Taiwan | 15 (100%) | 70 (65-82) | Breast (†) | Chemo-, radio-, hormonal therapy | On/post (>6 months since diagnosis) | Interviews | † | Content analysis | Explore fatigue experiences of older Taiwanese women. |
| Borneman et al.  (2012)  USA | 252 (†) | 60 (†) | Breast, lung, colon, prostate  (stage I-IV) | † | On/post (>2 months since diagnosis | Open-ended question-naire | † | Content analysis | Describe patients' perceptions of the causes, relief, related symptoms, meaning, and suffering secondary to fatigue. |
| Pertl et al.  (2014)  UK/Ireland | 73 (80%) | 51 (SD=11.5) | Breast, ovarian, lymphoma, leukaemia, prostate, colon, lung, sarcoma, gastric, cervical (†) | † | Post (M=3 years and 4 months since treatment (SD=22 months)) | Open-ended question-naire | Pluralist approach Willig (2012); realist approach, social constructionist discourse analytic approach | Inductive thematic analysis, critical and Foucauldian discourse analysis, | Examine factors that contribute to the absence of a discourse of fatigue. |
| Cordero et al.  (2015)  USA/Mexico | 39(†) | †(†) | Breast, prostate (early/late stage) | † | On/post (>6 months since treatment) | Focus groups | † | Content analysis | Examine how Latino patients experience fatigue (cancer/prior to diagnosis). |
| Watson & Van Kessel  (2016)  New Zealand, Australia, USA , Pacific Islands | 15 (73%) | 34 (21-54) | Breast, Hodgkin’s lymphoma, appendix, prostate, testicular, soft tissue sarcoma, osteo- sarcoma, non-Hodgkin’s lymphoma (†) | † | Post (>9 months since treatment) | Online blogs | † | Thematic analysis | Explore fatigue via online blogs, with a focus on their experiences and personal explanations of fatigue. |
| Hodge et al.  (2016)  USA | 132 (72%) | † (†) | † (†) | † | Post(†) | Focus groups | Grounded theory | Constructi-vist grounded theory techniques | Explore the experience of fatigue of American-Indian cancer survivors in the Southwest. |
| Hagan et al.  (2017)  USA | 47 (100%) | 57 (32-73) | Ovarian, fallopian or primary peritoneal (recurrent disease) | † | On/post(M=  45.10; SD=37.79 months since diagnosis) | Symptom care plan | † | Descriptive content analysis | Describe cancer patients’ goals and strategies for managing fatigue along with the process of individualizing both. |
| Levkovich et al.  (2017)  Israel | 13 (100%) | 52 (34-67) | Breast  (stage I-III) | Surgery,  chemo-therapy | Post (>1 month since treatment) | Interviews | Phenomenology | Conventio-nal content analysis | Explore the experience of fatigue, its effects, ways of coping and the role of family and social support. |

^†: missing items were not reported in studies^

**Table S3 CASP appraisal results**

| **Study** | **CASP score†**  **(reviewer 1)** | **CASP score†**  **(reviewer 2)** |
| --- | --- | --- |
| **Glaus et al. (1996)** | 27 | 28 |
| **Pearce & Richardson (1996)** | 21 | 21 |
| **Ream & Richardson (1997)** | 26 | 26 |
| **Holley (2000)** | 26 | 26 |
| **Ream et al. (2003)** | 21 | 20 |
| **Gledhill (2005)** | 18 | 18 |
| **Bennet et al. (2007)** | 20 | 22 |
| **Rosman (2009)** | 23 | 20 |
| **Tsai et al. (2010)** | 28 | 24 |
| **Borneman et al. (2012)** | 22 | 17 |
| **Pertl et al. (2014)** | 25 | 17 |
| **Cordero et al. (2015)** | 20 | 22 |
| **Watson & Van Kessel (2016)** | 26 | 22 |
| **Hodge et al. (2016)** | 23 | 18 |
| **Hagan et al. (2017)** | 25 | 16 |
| **Levkovich et al. (2017)** | 28 | 26 |

**^†:total sum scores of two authors in separate columns of 10 questions of CASP (question extensively addressed=3 points, partially addressed=2 points, and not addressed=1 point, minimum 10 points and maximum of 30 points. Q1: Was there a clear statement of the aims of the research; Q2: Is a qualitative methodology appropriate?; Q3: Was the research design appropriate to address the aims of the research; Q4: Was the recruitment strategy appropriate to the aims of the research; Q5: Was the data collected in a way that addressed the research issue; Q6: Has the relationship between researcher and participants been adequately considered?; Q7: Have ethical issues been taken into consideration?; Q8: Was the data analysis sufficiently rigorous?; Q9: Is there a clear statement of findings; Q10 How valuable is the research?^**

**Table S4 Overview of constructs (first-, second-, third-order) and theme (sub- and meta-)**

| **Third-order construct (meta-themes)** | **Merging categories (sub-themes)** | **Second-order construct**  **(examples)** | **First-order constructs (examples)** |
| --- | --- | --- | --- |
| 1. Embodied experience | Symptoms experienced in (whole) body | Bodily symptoms and sensations predominated (38–40,44,45) | *“Heavy limbs”* (39)  *“Legs like jelly or wobbly legs”* (39)  *“Feeling weak”* (19,31,45,46)  *“Paralysis”* (42) |
|  | Awareness of the body | Whole body experience (19,30,39–41,45,46)  Cognitive and affective symptoms as part of the body (19,30–32,38–40,43,45–47) | *“The body is worn-out”* (39,40,46)  *“Physically exhausted”* (19,39,45)  *“Body doesn’t want to go on”* (30)  *“Body cannot heal, nor can it function well”* (46)  *“The fatigue takes over every aspect of the body”* (19)  *“The feeling that the body dominated the mind” (39)*  *“The whole body is shattered” (40)*  *“The betrayal of the body and trapped in an old, sick body” (19,39,41)*  *“It’s like the body can’t … carry you. It’s a real weakness … a bad feeling. And it’s simply … like you don’t have any energy, no energy for anything. Nothing, like you’re … you’re like a zombie; you just sit there … like a couch potato”* F, 67, breast cancer (19) (p6).  *“The brain is out of function”* (30,40,41) |
|  | Interrelations of symptoms | Physical and mental symptoms spiral or vicious circle (37,39,40,45–47) | *“Fatigue is a physical experience but there is also something psychological; if one thinks of being a death candidate, it’s not cheering you up”* N/A (40) (p87). |
| 1. (Mis)Recognition | Social misrecognition | Invisibility (30,42–44,46,47) and difficulty to explain (30,32,37–40,43,44,46,47) | *“Because I look so much better than when I was having treatment, people think that I am back to prior fitness”* F, N/A (44) (p153). |
|  |  | Misunderstanding (30,43,44,46,47), conflict of needs and expectations (19,47), pressure to move on (44), lack of support friends and family (19,42,43,47) | *“My family is pretty good but they can easily forget how tired I can get as this is not obvious”* F, N/A (44) (p153). |
|  | Medical misrecognition | Lack of objective parameters (42,44) | *“No symptoms, no cause and no cure”* (44) |
|  |  | Lack of communication (44,45), lack of awareness (32,44) and low priority in management (32) | *“The least worrying side effect”* (32)*.*  *“Comes at the bottom of the list”* (32). |
|  |  | Lack of information after treatment (32,44,46) | “*I have not been told that I have fatigue but I seem to be living with it- it varies a lot but I do believe it interferes with my daily life and the sense of needing to ‘keep going’ because ‘it must be all in my head’ because I’ve not really had any diagnosis, and this makes me feel worse when I am tired, because I feel silly”* N/A (44) (p154). |
|  | Medical recognition | Information provision (32,43) | *‘‘This material has helped a lot, it has given me good advice and especially I felt reassured that fatigue was not absurd or unusual but that others suffer from it as well’’* (32) N/A (p105)*.* |
|  | Self-misrecognition | Lack of reporting (43,46,48)  Lack of information (19,30,44,46)  Pessimistic beliefs (30,31,43,45–47)  Embarrassment (19,39,43)  Gap between ‘life after illness’ and reality (19)  ‘Limbo between sickness and survivorship’ (44) | “*I’ve settled in my mind that I’m going to get worse; I expect it to happen gradually, and I expect one of the ways it will show is that I will be more tired than I am now”* N/A (37) (p114).  *“untreatable”*(45)*, “inevitable”* (43,45,47), *“sign of decline in health”* (30), *“it is a sign of not being successfully recovered from cancer or cancer recurrence”*(47), *“it indicates the body cannot heal nor function well”*(46), *“it elicits concerns about survival”* (31) or *“caused by the unknown”*(31). |
|  | Self-recognition | Recognition (39,41,42)  Seek for help (19,30–32,43–46,48)  ‘Illness with its own rights’ (39,42)  Optimistic beliefs (32,37) | *“Abnormal” and “pathological”* (39,41,42)  *“I don‘t think there’s going to be any problem in the future”* N/A (37) (p114).  *“It would be very helpful to have after care on a regular basis to help cope with fatigue. And also awareness campaign so people who don’t have cancer may be able to understand that cancer doesn’t end when chemo ends*” N/A (44) (p151). |
| 1. Small horizon | Obstacle in daily life | Unable to live a productive life (31,37,38,41,43)  Influences all kind of aspects of daily life (19,30,37,38,42,44). | *Reading* (39,41)*, getting out of bed* (19,31)*, moving* (42,45)*, walking* (39,40,45)*, climbing stairs* (39)*, carrying out household tasks* (38,39)*, praying* (30)*, working* (30,32,42)*, and socialising* (19,30–32,47) |
|  | Isolation and not belonging | Loneliness (31,39,44)  Wanted to be left alone and face fatigue by themselves (19,43,44) | *“Gradual submersion”, “drowning*”, and *“rising water”* (39) |
|  | Loss motivation and interest | Impact on motivation to do things (30,38,39,41)  Loss of interest in enjoyable things and in life in general (30,38,39,46) | *I don’t sleep well. I lie awake for hours at night, and in the morning, I drag myself out of bed, straggle around taking endless catnaps, living in a state of lethargy, where just watching the clouds go by is my only interest. No reading, no pleasure in doing anything whatsoever, especially all those activities I so loved before I became ill and tired… and in the evening I never feel like going to bed. On the other hand I just fall asleep when and where I shouldn’t in the day”* F, 62, leiomyosarcoma of uterus (39) (p299)*.* |
| 1. Role change | Family role | Impact on family system (19,30,31,37,41,43,46)  Dependency, others took over activities (19,31,39,43)  Stress and conflicts (19,43) | “*I let go, you know … it’s okay. It doesn’t mean anything. True, you were used to running things … at first, it’s some kind of contradiction, it’s … it’s a shock. You manage everything, then suddenly you feel … limited. Even your young child is bringing you a glass of water”* F, 43 breast cancer (19) (p6)*.* |
|  | Partner role | Impact on partner of being dependent (19,31); supportive (19), less supportive and even unsupportive (19,31) | “*I actually felt like I was being led like a little girl … whose father is leading her [by the hand*]” F, 43, breast cancer (19) (p7)*.*  *“He asked me if I could help him with something … I said—Me help you?! You need to help me! How can I help you? I can’t … it was really difficult”* F, 65, breast cancer (19) (p7). |
|  | Social role | Impact on social relationships (distress in social support network (19,30–32,38,40–44,46), social well-being disrupted (31), inability to attend community life (46)) | “*I’m the type of person, when someone’s ill or needy, or a friend needs something … I’m there right away … reporting for duty. Immediately. There’s no way that someone needs something and I won’t turn the world upside down to help them and … what happened actually, with my illness, is that … it’s as if everyone I’ve ever helped, everyone I’ve spent time with … came to repay me”* F, 41, breast cancer (19) (p7). |
|  | Work role | Impact on work (cut back on their workload, quit work, retire from work or prevent to return to work) (30,31,37,42,47) | *“I was determined to go back to work as soon as I possibly could. I mean I haven’t a particularly hard job, but then I did it for a week and just had to, just couldn’t do it anymore, and it really shocked me, that I felt so tired, so fatigued...so I think it would probably be quite handy to just make people aware of the fact that even though you are feeling quite well that the fatigue is going to get you, possibly will get you, and may well mean that you can’t work”* F, N/A (32) (p106). |
| 1. Loss of self | Feelings of loss | Lack of control (19,30–32,38,39,44),  Loss of confidence (39,41,44),  Worthlessness (31,41),  Indecisiveness (30,40),  Uncertainty in ability to cope with fatigue (19,30,31,38–40),  Loss of purpose in life and future (31,44),  Awareness of finite nature of life (39) | *“Hanging on to life by a thread of cotton”, “walking along the razor’s edge” or “along a tight rope”* N/A (39) (p304). |
|  | Identity change | No way back to ‘old self’ (37–39,44)  Active to inactive person (30,39,45)  Feeling an ‘old sick person’ inside (19,44,45) | *‘‘I was completely exhausted and in despair, living in a state of ‘‘absence’’, with regard to myself. My fatigue no longer served as an alert to the gravity of my condition. Rather, I came to tolerate it, unaware of the dangers due to my loss of lucidity. I felt ashamed of my powerlessness to face up to my fatigue, totally lost like a compass unable to find the North’’ F*, 62, (39) Leiomyosarcoma uterus (p310).  “*I’m obviously not the man I was”* N/A (37) (p114).  “*I feel like I’m in the body of an 80-year-old. That theoretically I know what my capabilities are, but … in reality … it’s different. It’s two different things. You know what you can do. But on the … on the other hand, you don’t. Now, it’s … exactly like an 80-year-old. Every single thing is ‘a project’. I cook, I need to sit down and rest. Ten minutes, yes? Prepare something, need to sit down and rest. These are things that … it’s like being trapped in the body of an 80-year-old”* F, 34, (19) breast cancer (p5). |
| 1. Regaining one’s footing | Struggle against CCRF | Fight it, continued their life roles, tried to fix it, distract themselves, concealed their CCRF (19,31,32,40,43–45,47,48) | “*I was very tired . . . I was tired, but my goal—my goal was to get through it as fast as I could. I pushed myself, I pushed myself, I pushed myself. Yeah, I was tired. I mean, I would be at work, I would have to put my head down or my sister used to work with me and I would tell her, ‘You know what? I’m just going to go take a nap.’ But I pushed myself, I pushed myself and I, I never gave up my heels”* F, N/A (45) (p11). |
|  | Adaptation to CCRF | Monitor activities to regain control (exercising (31,39,43,47,48), lifestyle changes (30,31,43,47,48), keeping a diary (32,48), building their lives on their fatigue experience (42)) | “*I have a very orderly and structured lifestyle. I don’t do anything which has not been planned. Every day I get up at 9 o’clock, I read, I do my shopping; I always eat at 1 p.m. before having a nap until 4, then I watch a bit of TV, I eat at 6, lie down until 8, watch TV with my husband, then go back to bed at 10. It’s a very regular life, but it suits me*” F, 62, Non-Hodgkin lymphoma (42) *(*p32). |
|  |  | Monitor CCRF (being vigilant, recognising and setting boundaries, listening to personals needs and listening to their bodies) (19,39,47,48) | *“Fatigue taught me to listen to my body which is now so fragile”* F, 48, Acute Lymphoblastic Leukaemia (39) (p310). |
|  |  | Pacing activities (stopping doing activities: having a break (40), sitting or lying down to rest or sleep (31,39,40,42,45,47,48), resting and waiting for it to pass (19,31,37,39,43) and avoiding physical straining activities (30,42). | “*If I’m more tired, it’s not the end of the world, so I’ll rest and do less. It’s not the end of the world and it’s possible to live at a slower pace than I was used to doing”* F, 60, breast cancer (19) (p6). |
|  |  | Re-shaped their lives: living their life day by day (38,43), planning (19,30,42,43,48), prioritising (19,30,43,48), reorganising life and work (19,30,42,43,47), balancing activities (19,46). | “*I was a very active person, and I’ve gone from a very active life to . . . if I overexert, I’ll tire myself out. I can’t work for a couple reasons. First, the bank won’t let me come back without a full medical release. Second, I wouldn’t be able to do the job. You just have to adjust your lifestyle, and try to get the control back”* M, 51, gastroesophageal cancer (30) (p92). |
|  | Acceptance of CCRF | Internalizing current change in situation, trying to see positive aspects and finding new balance, change attitude (19,31,37,43,47,48) | “*It’s all about attitude really ... in altering what you can, accepting what you can’t”* N/A (37) (p114). |
|  |  | Explore and reframe meaning (naming or assigning a cause to CCRF to give it place in their life (30,31,39,44), set goals and visualise strategies to overcome fatigue, legitimise fatigue and get support from family members (32,42–44,46,48)) | *“It is a matter of legitimisation to experience fatigue as a usual experience”* N/A (32) (p105)*.* |
| Relations of meta-themes (embodied experience, (mis)recognition, small horizon, role change, loss of self, regaining one’s footing) |  |  | *“I am at the age where people around me go out, party and have fun all the time. That’s what college students do! ….. but I can’t. Nobody understands that”* N/A (47) (p4) (role change, small horizon and misrecognition). |
|  |  |  | *“Functioning … almost not at all. But with that, I didn’t give up. I’m a person who doesn’t give up … so I sat more, rested more … but I didn’t give up.”* F, 41, breast cancer (19) (p6) (small horizon and regaining one’s footing). |
|  |  |  | *“I feel so tired. I don’t know why I worry so much. More than one hundred days of worries and lack of sleeping my body condition get worse and worse…some of my children are not married yet; this means my duties are still unfulfilled”* F, N/A, breast cancer (43) (p870) (embodied experience and regaining one’s footing). |
